# Genome-Wide Identification and Expression Analysis of the *DMP* and *MTL* Genes in Sweetpotato (*Ipomoea batatas* L.)

**DOI:** 10.3390/genes15030354

**Published:** 2024-03-12

**Authors:** Zhiyuan Pan, Zongyun Li, Yonghua Han, Jian Sun

**Affiliations:** 1 Institute of Integrative Plant Biology, School of Life Sciences, Jiangsu Normal University, Xuzhou 221116, China; 1020220014@jsnu.edu.cn (Z.P.); zongyunli@jsnu.edu.cn (Z.L.); 2 Jiangsu Key Laboratory of Phylogenomics & Comparative Genomics, School of Life Sciences, Jiangsu Normal University, Xuzhou 221116, China

**Keywords:** sweetpotato, double haploid, haploid induction, *DMP*, *MTL*

## Abstract

Sweetpotato (*Ipomoea batatas* L.) is a strategic crop with both economic and energy value. However, improving sweetpotato varieties through traditional breeding approaches can be a time-consuming and labor-intensive process due to the complex genetic nature of sweetpotato as a hexaploid species (2n = 6x = 90). Double haploid (DH) breeding, based on in vivo haploid induction, provides a new approach for rapid breeding of crops. The success of haploid induction can be achieved by manipulating specific genes. Two of the most critical genes, *DMP* (DUF679 membrane proteins) and *MTL* (MATRILINEAL), have been shown to induce haploid production in several species. Here, we identified and characterized *DMP* and *MTL* genes in sweetpotato using gene family analysis. In this study, we identified 5 *IbDMPs* and 25 *IbpPLAs*. *IbDMP5* and *IbPLAIIs* (*IbPLAIIκ*, *IbPLAIIλ*, and *IbPLAIIμ*) were identified as potential haploid induction (HI) genes in sweetpotato. These results provide valuable information for the identification and potential function of HI genes in sweetpotato and provide ideas for the breeding of DH lines.

## 1. Introduction

Sweetpotato is a globally significant economic and energy crop, known for its high nutritional content and adaptability to diverse environmental conditions [1]. However, the breeding of sweetpotato encounters significant challenges that impede the advancement of superior varieties. One of the main obstacles is the complex genetic properties of sweetpotato (2n = 6x = 90). Sweetpotato presents challenges for trait selection due to its high ploidy, heterozygosity, chromosome count, and incompatible self-breeding [2]. To accelerate the development of improved cultivars, researchers have turned to innovative techniques such as haploid induction combined with gene editing to streamline the breeding process [3].

Haploid induction (HI), which involves the production of haploid plants, offers significant advantages in achieving homozygosity quickly, allowing for the identification of desirable traits efficiently [4]. The success of haploid induction depends on the manipulation of specific genes that play crucial roles in the process. In this context, several genes have been identified and studied extensively for their involvement in haploid induction. Notably, the genes *DMP* [5] and *MTL/ZmPLA1/NLD*(MATRILINEAL/*Zea mays* PHOSPHOLIPASE A1/NOT LIKE DAD) [6,7,8], along with their homologs, have emerged as key players in haploid induction in different crop species.

One of the essential players in haploid induction is the DOMAIN OF UNKNOWN FUNCTION 679 membrane protein (*DMP*) [9]. *DMP* genes are conserved in dicots and have been shown to be involved in gamete fusion during double fertilization [10]. The Cas9/gRNA-mediated successful creation of HI lines by knockdown of *DMP* homologs in a variety of plants, including maize [11], tomato [12], potato [13], watermelon [14], *Brassica napus*, and *Nicotiana tabacum* [15], suggests their potential roles in the haploid induction process.

The *MTL/ZmPLA1/NLD* gene has been associated with the haploid induction process in maize [6,7,8]. *MTL/ZmPLA1/NLD* encodes a pollen-specific phospholipase, regulating the formation and development of maize pollen, as well as the process of pollen tube elongation. Inducing a knockdown of the *MTL/ZmPLA1/NLD* genes via gene-editing technology triggers the production of maize haploid seeds, propelling haploid breeding into a phase of rapid development. Similarly, employing gene-editing techniques for the knockout of *OsMATL* in rice, a homologous gene to maize *ZmMTL*, induces the generation of haploid seeds in rice [16]. Within the heterozygous hexaploid common wheat, a mutant of *TaPLA* (Wheat patatin-related phospholipase A) was successfully derived through homologous gene cloning and gene editing, capable of yielding approximately 2–3% haploid kernels without compromising the growth and development of wheat or of pollen viability [17]. These findings portend potential advancements in haploid breeding for sweetpotato with heterozygous hexaploids. Particularly noteworthy, recent research has demonstrated that the loss of function in the stamen-expressed *AtPLAIIγ* can instigate maternal haploid induction, marking the initial instance where the *ZmPLA1* homologous gene induces haploid induction in dicotyledonous plants [18]. Explorations of the homologous gene in sweetpotato may potentially reveal novel targets for augmenting haploid induction efficiency.

In summary, haploid induction combined with gene editing offers tremendous potential for accelerating sweetpotato breeding programs and developing improved cultivars with desirable traits. Comprehending pivotal genes like *DMP* and *MTL/ZmPLA1-/NLD* is crucial for the accurate screening and identification of haploid induction genes in sweetpotato. Although advancements have been made in mapping the sweetpotato genome, investigations into genes associated with haploid breeding remain scarce. In this study, multiple potential genes for haploid induction from two gene families were identified for the first time in sweetpotato, and the genomic resources of sweetpotato were optimized and organized. Potential haploid-inducing genes (*IbDMP* and *IbMTL*) were successfully identified in sweetpotato through a combination of bioinformatics analysis methods and tissue differential analysis. This discovery lays the groundwork for the development of efficient and streamlined breeding strategies for this economically significant crop.

## 2. Materials and Methods

### 2.1. Plant Materials

Plant samples were collected from the experimental field under natural lighting conditions. Roots were undifferentiated; stems were harvested 10–15 cm below the terminal bud; and leaves were obtained from the 3rd–4th piece below the terminal bud. Flowers were dissected into various parts based on their composition, including calyx, petals, filaments, anthers, ovary, and stigma. Immature and mature anthers were selected based on bud size (Figure S1). Microscopic images of pollen microstructure were acquired (Figure S2). Approximately 60 flowers were collected.

### 2.2. Identification of the IbDMP and IbpPLA Protein Family Members in Sweetpotato

The Ipomoea Genome Hub database (https://sweetpotao.com/download_genome.html, accessed on on 10 June 2023) was utilized to acquire the entire genome sequence and GFF annotation data of sweetpotato (Ipomoea batatas, Taizhong6). Nucleotide and protein sequences of DMPs and pPLAs from *Arabidopsis* (model plants, dicotyledonous plants), *Oryza sativa* (monocotyledonous crops), and *Zm* (monocotyledonous crops) were retrieved from TAIR (https://www.arabidopsis.org/, accessed on 12 June 2023), the Rice Genome Annotation Project (http://rice.uga.edu/, accessed on 12 June 2023), and MaizeGDB (https://www.maizegdb.org/, accessed on 10 June 2023), respectively. The sequences of *DMP* and *MTL* genes from other species were obtained from Ensembl Plants (http://plants.ensembl.org/index.html, accessed on 12 June 2023). All genes information is listed in Supplementary Table S1. Subsequently, we conducted a blast of the homologous sequence against the sweetpotato genome database using TBtools [19] with an e-value of ≤1 × 10^−5^, as described in a previous report [20]. Additionally, the Hidden Markov Model (HMM) file was obtained from the Pfam database [21] (http://pfam.xfam.org/, accessed on 15 June 2023) and used to search for all sequences in sweetpotato via HMMER 3.2.1 [22] (e-value < 0.01). Subsequently, we combined the two candidate sets (Figure S3). The NCBI CD-Search tool [23] (https://www.ncbi.nlm.nih.gov/cdd, accessed on 15 June 2023) and the PROSITE database (https://prosite.expasy.org/, accessed on 15 June 2023) [24] were employed to filter out proteins.

### 2.3. Protein Properties Prediction and Chromosomal Distribution of Genes

The ExPASy tools (http://expasy.org/, accessed on 26 June 2023) and WoLF PSORT (https://www.genscript.com/wolf-psort.html, accessed on 26 June 2023) were used to predict the physical and chemical characteristics of protein properties, including molecular weight (MW), protein length based on the number of amino acids (aa), theoretical isoelectric point (pI), total average hydrophilicity (GRAVY), and subcellular locations. Subsequently, we conducted a detailed analysis of the tertiary structure of proteins. Homologous modeling of HI genes from various species, as well as those identified in sweetpotato, was performed using the online platform (https://swissmodel.expasy.org/interactive, accessed on 20 July 2023).The chromosome location of each gene was retrieved from the GFF3 file of the Ipomoea Genome Hub and then visualized on sweetpotato chromosomes using TBtools. All gene information is listed in Supplementary Table S2.

### 2.4. Amino Acid Sequence Comparison and Phylogenetic Analysis

The unrooted phylogenetic tree was constructed using MEGA11 [25] based on the amino acid sequences of sweetpotato, *Arabidopsis*, rice, and other species. All the protein sequences were aligned by ClustalW [26] with the default settings, and conserved sequences were shaded using GeneDoc. We constructed two different types of phylogenetic trees: a neighbor-joining (NJ) tree with a bootstrap method of 2000 replicates and a Poisson model with default parameters as well as a maximum likelihood phylogenetic tree (ML) with the best evolutionary model of JTT + G + I and a bootstrap value of 1000 with partial deletions. ChiPlot [27] was used to construct the phylogenetic tree.

### 2.5. Analysis of Motif, Gene Structure, and Conserved Domain

The MEME tool [28] (https://meme-suite.org/meme/tools/meme, accessed on 26 July 2023) was used to analyze the conserved Motifs of all obtained sequences. The NCBI Conserved Structural Domains Database (CDD) (https://www.ncbi.nlm.nih.gov/Structure/cdd/wrpsb.cgi, accessed on 26 July 2023) was used to identify the type and location of structural domains. Then, we used the TBtools software 1.098 to visualize the exon–intron structure, conserved Motifs, and conserved domains of all protein sequences.

### 2.6. Analysis of the Gene Promoter in Sweetpotato

The 2000 bp DNA sequence upstream of the initiation codon (ATG) of each gene was obtained from the sweetpotato genome and thensubmitted to the PlantCARE database [29] (https://bioinformatics.psb.ugent.be/webtools/plantcare/html/, accessed on on 28 July 2023) for prediction and analysis of *cis*-acting regulatory elements related to phytohormones, plant growth and development, and abiotic stress in promoter regions of genes. All gene information is listed in Supplementary Table S3.

### 2.7. Gene Interaction Network of the Proteins

STRING database [30] (https://string-db.org/, accessed on 30 July 2023) was employed to analyze protein interactions based on Arabidopsis orthologous proteins, with a confidence parameter set at a threshold of at least 0.7 [31].

### 2.8. qRT–PCR of Genes

Total RNA was extracted from various tissues, including root, stem, leaf, petal, anther (both immature and mature), filament, stigma, ovary, and sepal, of elite sweetpotato cultivar Xuzishu 8 using TRIzol reagent (Takara Bio Inc., Beijing, China). The collected samples were sorted by tissue type for RNA extraction: roots, stems, and leaves were obtained from three flowering Xuzishu 8 plants; 60 flowers were collected and divided into various tissues based on bud size. Subsequently, the samples were pooled together. Quantitative reverse transcription–polymerase chain reaction (qRT–PCR) was performed following the established protocol [32]. The qRT–PCR primer sequences for the genes are provided in Supplementary Table S4. The relative gene expression level was normalized to the reference gene (*IbUBI*) and calculated using the 2^−ΔΔCt^ method.

## 3. Results

### 3.1. Identification of the IbDMP and IbpPLA Protein Family Members in Sweetpotato

**Figure 1:**
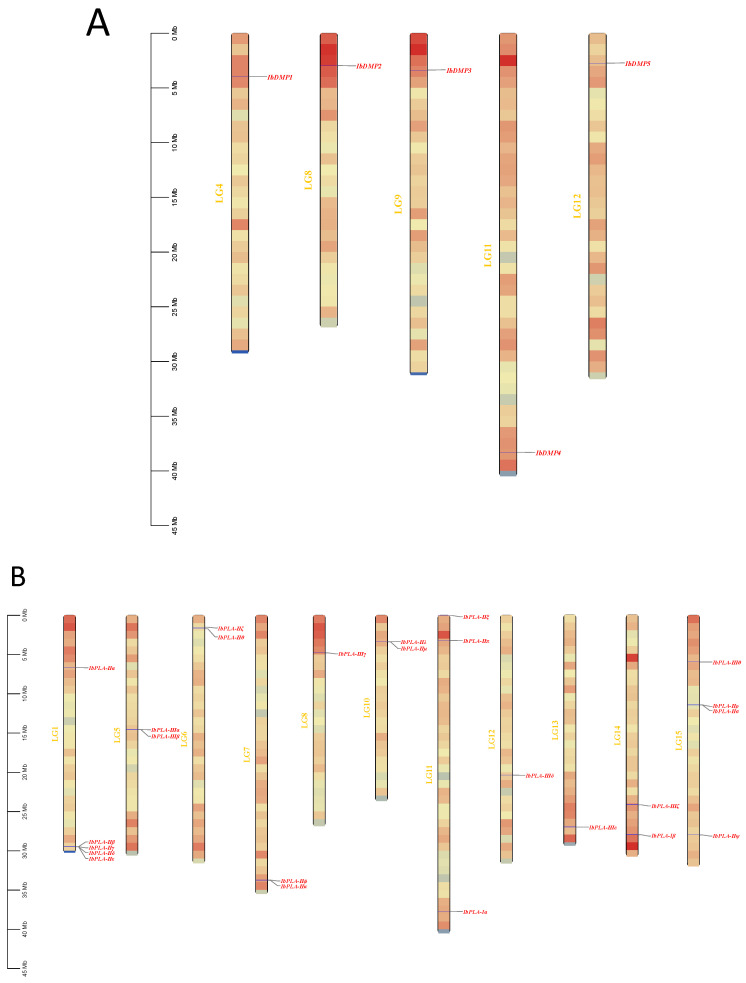
Chromosomal localization and distribution of *IbDMPs* (**A**) and *IbpPLAs* (**B**). All members were named according to the location order of sweetpotato chromosomes from top to bottom.

To identify all members of IbDMPs and IbpPLAs in sweetpotato (Ipomoea batatas, Taizhong6), we conducted a BLASTP search using the protein sequences of these protein family members in *Arabidopsis*, *Oryza sativa*, and *Zea mays* [33,34,35,36,37] as queries. Additionally, the Hidden Markov Model (HMM) file from the protein family (Pfam) database was used to query these protein members using HMMER 3.2.1 (e-value < 0.01; the protein families queried were DMPs (PF05078) and pPLAs (PF01734)). Subsequently, the two candidate sets were combined (Figure S3), and the proteins were excluded using the NCBI CD-Search tool and the PROSITE database. Consequently, a total of 5 DMP protein sequences and 25 pPLA protein sequences were identified in sweetpotato. Finally, all members were named according to the location order of sweetpotato chromosomes from top to bottom, as shown in Figure 1.

### 3.2. Characteristics of the IbDMP and IbpPLA Protein Family Members in Sweetpotato

We conducted an analysis of the fundamental characteristics of IbDMPs and IbpPLAs using sequences from the sweetpotato genome. IbDMPs displayed putative protein lengths ranging from 209aa to 846aa, MWs from 22.875 kDa to 95.615 kDa, and pIs from 4.82 to 9.71. GRAVY scores were mostly above 0, with the exception of IbDMP1. In addition, all proteins, except IbDMP5, were deemed unstable. The subcellular localization prediction suggests a wide distribution within this protein family. IbPLAs have putative protein lengths ranging from 292aa to 1408aa, MWs from 32.779 kDa to 153.971 kDa, and pIs from 4.83 to 9.31. These proteins are characterized as hydrophilic, and the majority are stable. The subcellular localization prediction indicates a broad intracellular distribution for this family. Table S2 provides comprehensive information on the characterization of all members of the two gene families.

### 3.3. Phylogenetic Analysis of the IbDMP and IbpPLA Protein Family Members in Sweetpotato

**Figure 2:**
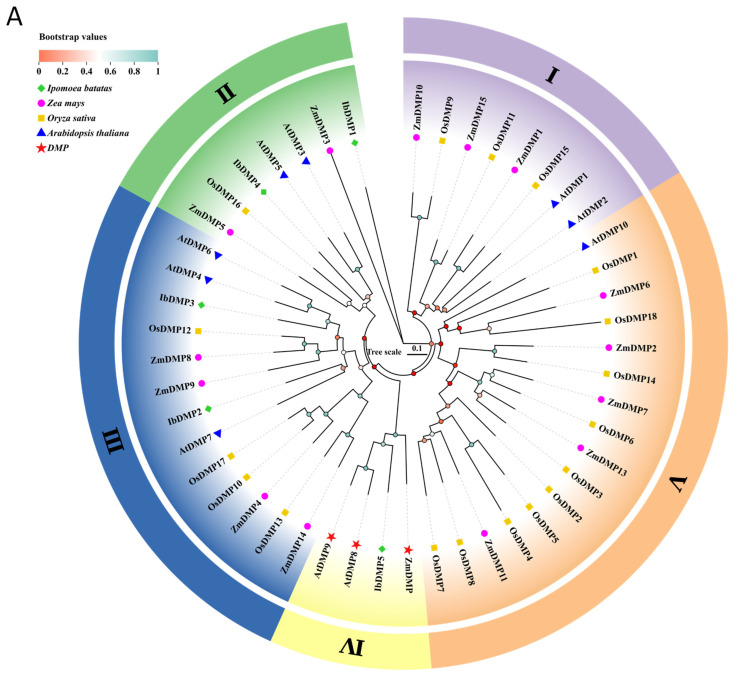

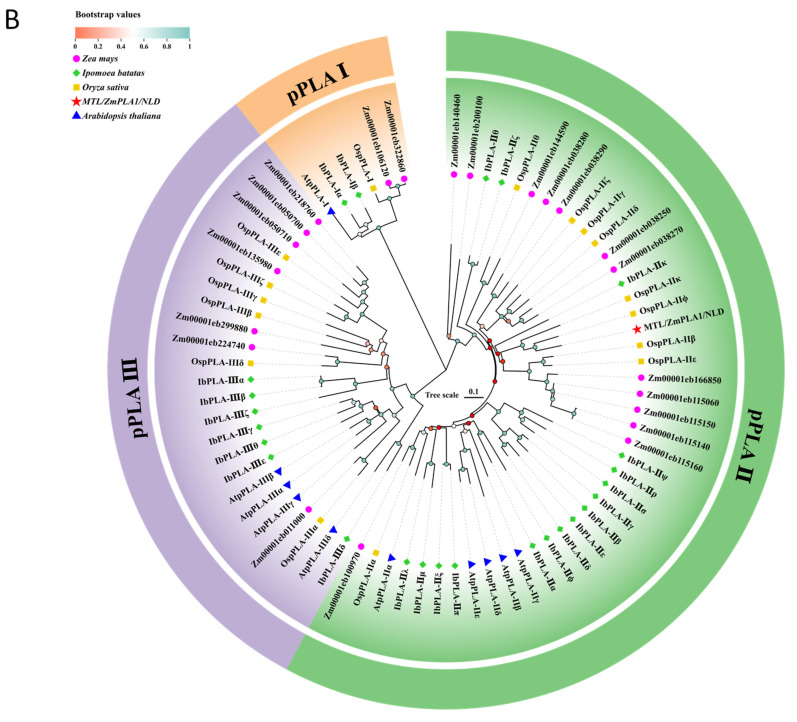
The NJ phylogenetic tree of DMPs (**A**) and pPLAs (**B**). The phylogenetic tree was constructed using the neighbor-joining method implemented in MEGA11 software 11.0.11. Bootstrap values are indicated by differently colored circles in the central region of the tree branches. Various species are denoted by different colored shapes: blue triangle for *Arabidopsis*, green rhombus for *Ib*, yellow square for *Os*, powdered circle for *Zm*, and red star for haploid-inducible genes. Different subfamily groups are highlighted with distinct colors and labeled in the outer ring. I–V represent subfamilies of *DMPs*.

We utilized MEGA11 to construct a neighbor-joining tree to investigate the evolutionary relationships among IbDMPs and IbpPLAs in sweetpotato and other species. Regarding DMPs, 5 IbDMPs, 10 AtDMPs, 18 OsDMPs, and 15 ZmDMPs were classified into five subfamilies (subfamily I, II, III, IV, and V) based on their evolutionary distance (Figure 2A). The IbDMPs comprised five members distributed across three subfamilies (II with 2, III with 2, and IV with 1). Concerning pPLAs, 74 pPLA proteins from these four DMP species were categorized into three groups (pPLAⅠ, pPLAⅡ, and pPLAⅢ) based on the phylogenetic tree (Figure 2B). Notably, the pPLAⅡ subfamily had the highest number of IbpPLA genes, totaling 16 members. The pPLAⅠ subfamily contained only 2 members, while the pPLAⅢ subfamily included 7 members.

### 3.4. Conserved Motif and Domain Analysis of the IbDMP and IbpPLA Protein Family Members in Sweetpotato

**Figure 3:**
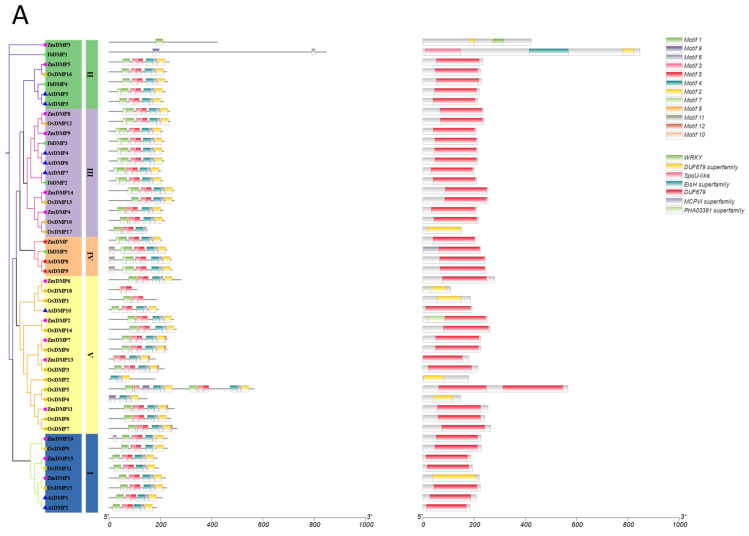

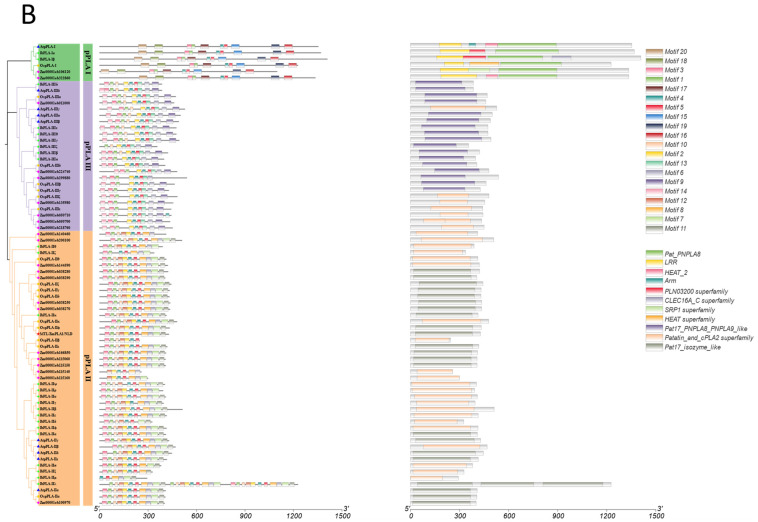
Conserved Motif and domain analysis of DMPs (**A**) and pPLAs (**B**). Individual plots included phylogenetic trees, Motifs (top 20 Motifs), and domain analysis. I–V represent subfamilies of *DMPs.* Coordinate axes are in units of: aa.

To explore the similarity of conserved Motifs and domains within the same protein family across different species, we utilized the MEME program and the NCBI CD-Search tool to identify these elements. Employing the phylogenetic tree, we aligned the conserved Motifs and domains of DMP and pPLA protein family members in sweetpotato, rice, maize, or *Arabidopsis* (Figure 3). Specifically, we characterized the first 20 conserved Motifs of each histone protein in sweetpotato and *Arabidopsis*.

Subsequently, based on the phylogenetic tree, we analyzed the top 10 conserved Motifs of the DMP family in four different species: sweetpotato, rice, *Arabidopsis*, and maize. The results demonstrated that the DMP family is highly conserved among these four species. All members of subfamily II, except IbDMP1 and ZMDMP3, exhibit Motif1, Motif2, and Motif3, and all feature the DUF679 structural domain (Figure 3A).

Similarly, we analyzed the first 20 conserved Motifs of the pPLA family members in four species. There are noticeable differences in the structure among different subfamilies in the pPLA family: pPLAI family members have the longest protein length and the smallest number of proteins with the LRR + Pat_PNPLA8 domain; pPLAII has the greatest number of subfamily members and is relatively conserved; and the pPLAIII subfamily structure is similar to that of pPLAII subfamily but has a Motif9 structure and a Pat17_PNPLA8_PNPLA9-like domain (Figure 3B). The maize HI gene MTL/ZmPLA1/NLD belongs to the pPLAII subfamily.

### 3.5. Identification of Potential HI Genes in Sweetpotato

**Figure 4:**
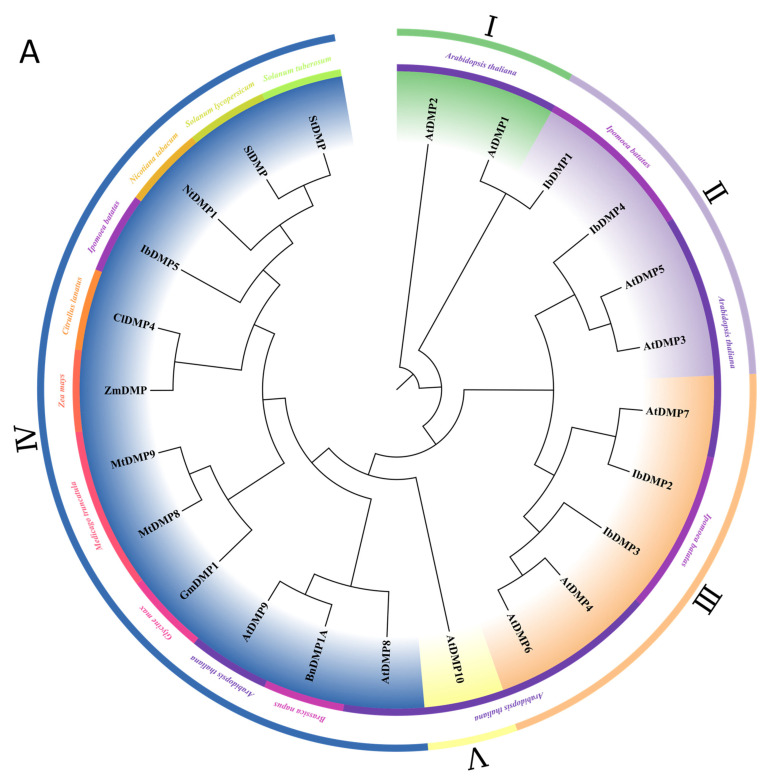

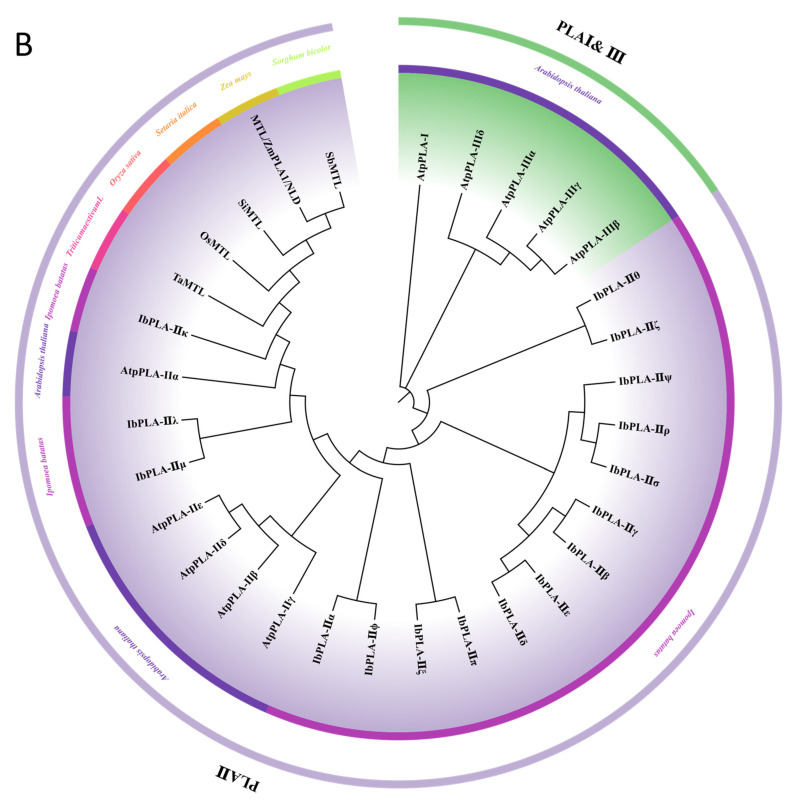
The ML phylogenetic tree of potential haploid inducer genes. (**A**) Involves *DMP* and its homologous genes. I–V represent subfamilies of *DMPs*. (**B**) Comprises *MTL* and its homologous genes. The phylogenetic tree was constructed using MEGA11 with the maximum likelihood method. The outer ring is color-coded to represent different subfamilies, and the inner ring is color-coded to represent different species.

**Figure 5:**
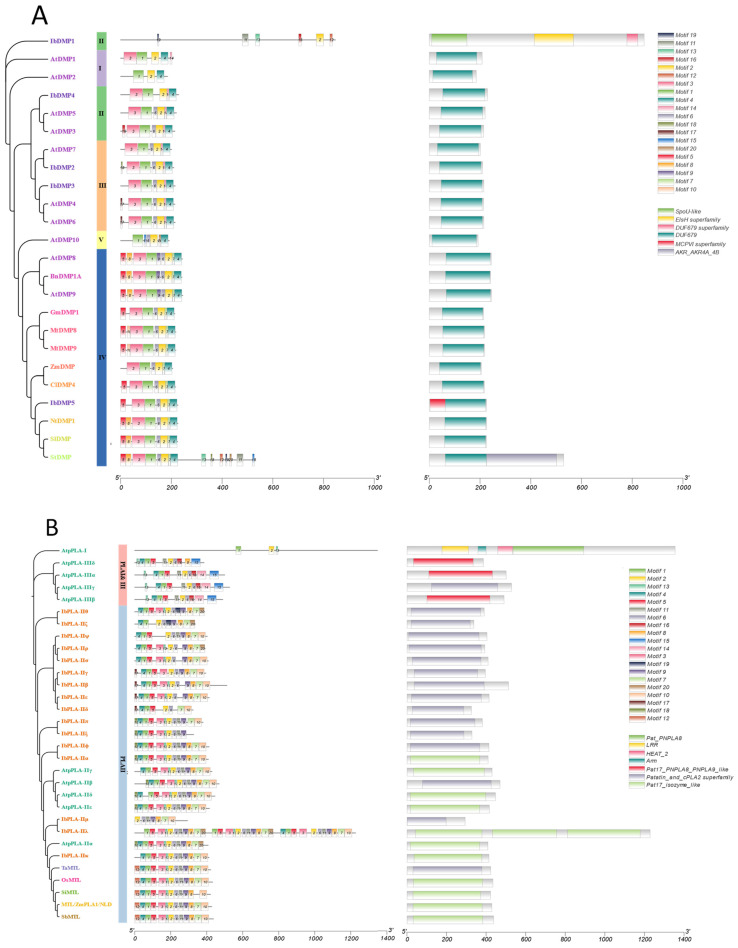
Conserved Motif and domain analysis of potential haploid inducer genes. (**A**) Contains *DMP* and its homologous genes. I–V represent subfamilies of *DMPs*. (**B**) Contains *MTL* and its homologous genes. Individual plots consisted of phylogenetic trees, Motifs (top 20 Motifs), and domain analysis. Different species are displayed with different color traits in the branches of the systematic tree. Coordinate axes are in units of: aa.

To further identify HI genes in the two gene families, we aligned the amino acid sequences of reported or homologous HI genes from other species (Figure S4) and constructed maximum likelihood (ML) trees (Figure 4). We calculated the model score for all protein sequences and selected the most suitable JTT + G + I model using the maximum likelihood method. The phylogenetic tree was then drawn using ChiPlot. Concurrently, we conducted a detailed analysis of the conserved Motifs and domains of the proteins (Figure 5).

Given that there are only 5 *DMP* genes in sweetpotato, we utilized all members of the *AtDMPs*, *IbDMPs*, and other *DMP* genes (*BnDMP1A* and *NtDMP1* [15], *ZmDMP* [5], *GmDMP1* [38], *MtDMP8* and *MtDMP9* [39], *ClDMP4* [40], *SlDMP* [12], and *StDMP* [13]) to construct a phylogenetic tree and perform a conserved structure analysis (Figure 4A). IbDMP5 shows higher homology with previously reported DMP proteins that are associated with haploid induction. With the exception of IbDMP1, the remaining four DMPs exhibit high conservation, all featuring a conserved structure of Motif3 + Motif1 + Motif6 + Motif4 with a DUF679 domain (Figure 5A). Amino acid sequence analysis indicates that the IbDMP2–IbDMP5 sequences are highly similar and may have analogous functions (Figure S4A).

The *MTL* genes associated with haploid induction that have been reported belong to the *pPLAII* family, including monocots such as maize [41], rice [16], and millet [42] and dicots such as *Arabidopsis* [18]. Analysis of the *pPLAs*, encompassing *IbpPLAs* and *AtpPLAs*, revealed that *IbPLAIIκ*, *IbPLAIIλ*, and *IbPLAIIμ* exhibited high homology with *ZmMTL* (Figure 4B). The results of the structural analysis showed that the majority of pPLAII members had a Motif4 + Motif1 + Motif5 + Motif3 + Motif13 + Motif2 + Motif6 + Motif11 + Motif9 + Motif8 + Motif7 + Motif10 structure (Figure 5B). This suggests that there are conserved functional regions within the *pPLAII* subfamily (Figure S4B).

### 3.6. Gene Structure and Promoter Region Cis-Acting Regulatory Elements Analysis

**Figure 6:**
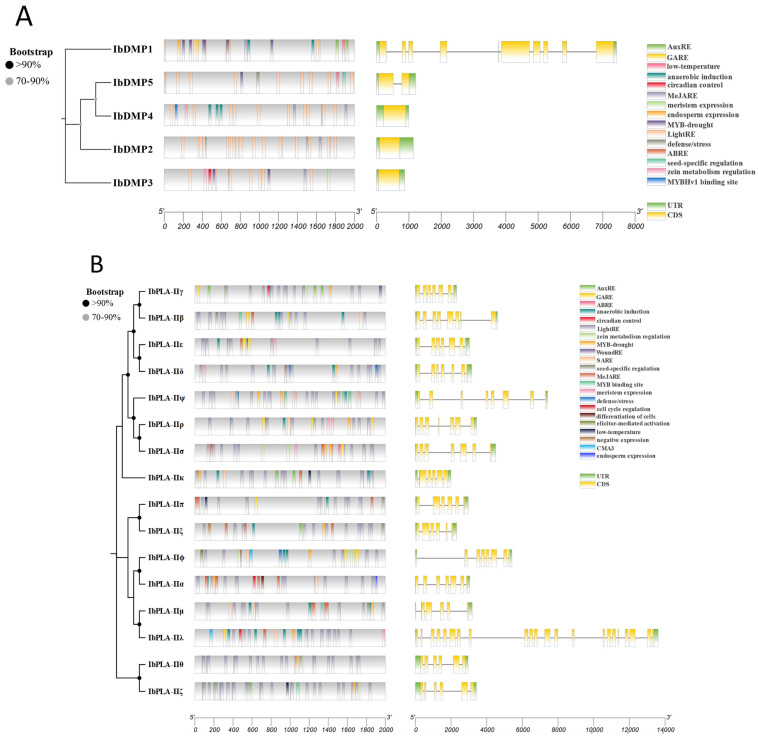
Gene structure and promoter region *cis*-acting regulatory elements analysis of potential haploid inducer genes. (**A**) Investigating promoter elements and gene structure of *IbDMPs*. (**B**) Examining IbpPLA II subfamily promoter elements and gene structure. Individual plots included phylogenetic trees, gene structure, and analysis of *cis*-acting regulatory elements in the promoter region. Bootstrap values are depicted with gray circles in the middle section of the system tree branches. Coordinate axes are in units of: bp.

To gain a deeper understanding of the potential traits of HI genes identified in sweetpotato, we examined gene structures and *cis* elements (Figure 6). Simultaneously, we constructed ML trees to elucidate the evolutionary relationships among members of the same family.

The ML tree indicates that *DMP2*, *DMP4*, and *DMP5* share a more recent evolutionary relationship. In terms of gene structure, *DMP1* exhibited 12 exons and 9 introns; *DMP5* had 4 exons and 1 intron; and *DMP2*, *DMP3*, and *DMP4* had 3 exons with no introns. *IbDMPs* encompassed 15 primary homeopathic regulatory elements, predominantly associated with light response (49.5%), hormone regulation (21.3%), adversity stress (19.5%), and growth and development (9.7%) (Figure 6A, Supplementary Table S3). Notably, *IbDMP5* possesses a seed-specific regulation element.

The *IbPLAII* comprises a total of 16 members, each exhibiting a specific number of exons and introns, along with 22 *cis*-acting regulatory elements (Figure 6B, Supplementary Table S3). Half of these elements are associated with light response, with approximately 6.3% linked to growth and development.

### 3.7. Protein Tertiary Structure and Potential Regulatory Network Analysis

**Figure 7:**
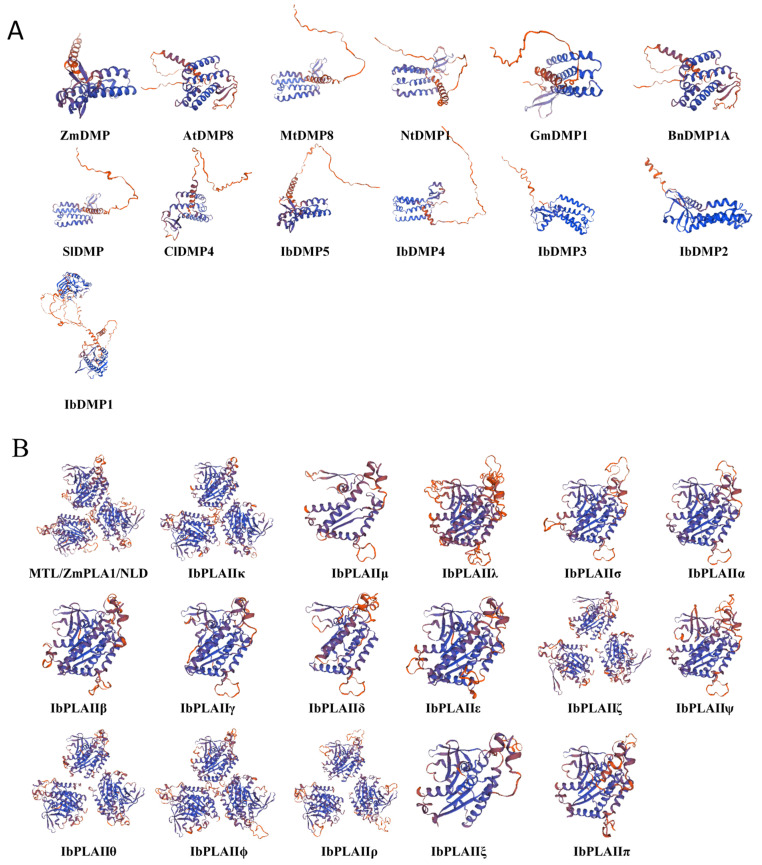
Diagram of tertiary structure pattern of potential haploid inducer genes in sweetpotato. (**A**) Tertiary structural modeling of IbDMPs and ZmDMP cognate proteins. (**B**) Tertiary structural modeling of IbpPLA II subfamily and ZmMTL/ZmPLA1/NLD cognate proteins.

**Figure 8:**
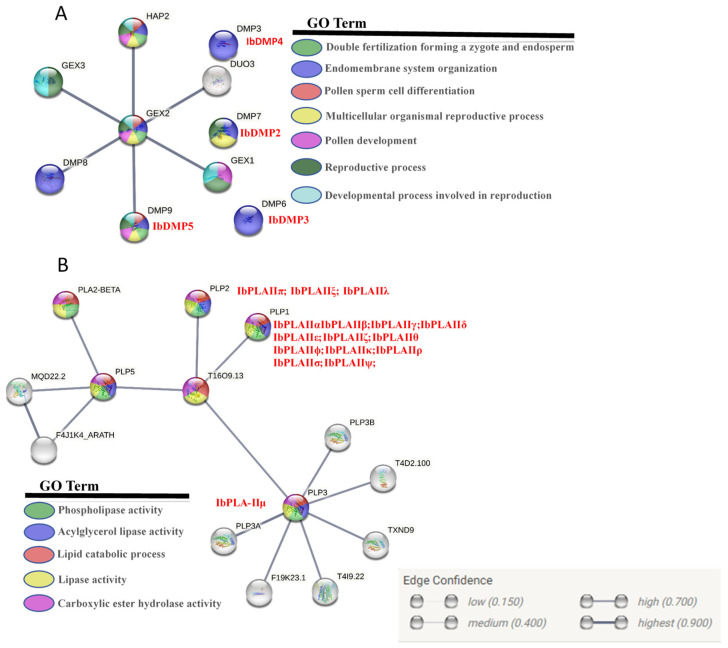
STRINGdb protein–protein interaction diagram. Revealing the potential functions of haploid-induced genes based on their homology to (**A**) thaliana proteins and their interactions. Colored nodes are those enriched with a Gene Ontology (GO) term with an FDR < 0.01. Network nodes represent proteins, and lines represent protein–protein associations. The thickness of lines represents the interaction strength. (**A**) IbDMPs potential protein regulatory networks and GO enrichment analysis. (**B**) IbpPLA II subfamily potential protein regulatory networks and GO enrichment analysis.

We conducted a detailed analysis of the tertiary structure of proteins and the potential protein regulatory network of candidate HI genes in sweetpotato. Subsequently, the tertiary structure pattern diagrams for the proteins were generated (Figure 7). At the same time, the regulatory network of haploid-inducible genes in sweetpotato was analyzed by constructing it using the STRING database to analyze the functions of related proteins (Figure 8).

The DMP family exhibited relative conservation in monocots and dicots, and the three-dimensional protein structure indicated that, with the exception of IbDM1, the remaining IbDMP members shared similarities with reported homologous genes associated with HI function (Figure 7A). As illustrated in Figure 8A, the IbDMP family members did not show interactions among themselves but were implicated in the reproductive development of sweetpotato. Notably, IbDMP5 forms a protein interaction network with GEX2 and HAP2 (score of 0.9), contributing to processes such as pollen sperm cell differentiation (GO:0048235), double fertilization forming a zygote and endosperm (GO:0009567), and pollen development (GO:0009555). In addition, IbDMPs are involved in the endomembrane system organization (GO:0048235) process and may contribute to the regulation of sweetpotato reproductive development through the endomembrane system.

IbPLAII exhibits a higher similarity with ZmMTL, sharing spatial conformations like α-helix, β-fold, and random coiling, albeit with some differences in quantity (Figure 7B). The IbPLAII family primarily engages in the metabolism of plant lipids, including processes such as the lipid catabolic process (GO:0016042), phospholipase activity (GO:0004620), and lipase activity (GO:0016298) (Figure 8B).

### 3.8. Tissue Expression Patterns Analysis of Potential HI Genes

**Figure 9:**
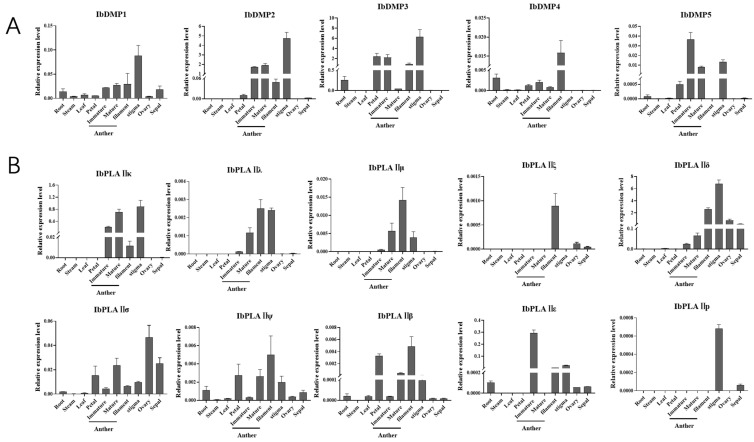
Tissue expression patterns analysis of potential haploid inducer genes in sweetpotato. (**A**) Tissue expression patterns of IbDMPs. (**B**) Tissue expression patterns of selected members of the IbpPLA II subfamily. Using qRT–PCR, tissue-specific expression was examined in 10 organs, including roots, stems, leaves, petals, anthers (both immature and mature), filaments, stigmas, ovaries, and sepals. Error bars represent standard deviations of the means of three technical replicates for each sample (*n* = 3).

HI genes typically exhibit high expression in reproductive organs [43]. To further validate potential HI gene expression patterns in sweetpotato, we selected 10 organs, including roots, stems, leaves, petals, anthers (both immature and mature), filaments, stigmas, ovaries, and sepals, for qRT–PCR experiments (Figure 9 and Figure S7).

*IbDMPs* showed pronounced expression in reproductive tissues. Specifically, *IbDMP1*, *IbDMP2*, and *IbDMP3* showed peak expression in the stigma; *IbDMP4* reached its highest level in the filament; and *IbDMP5* exhibited maximum expression in immature anthers (Figure 9A).

Expression patterns of the IbPLAII subfamily differed among the 10 tissues (Figure 9B). *IbPLAIIκ*, *IbPLAIIλ*, *IbPLAIIμ*, *IbPLAIIρ*, and *IbPLAIIξ* exhibited expression exclusively in reproductive tissues. *IbPLAIIα*, *IbPLAIIθ*, and *IbPLAIIφ* showed high expression in vegetative tissues. *IbPLAIIα* and *IbPLAIIφ* exhibited the highest expression in roots, while *IbPLAIIθ* demonstrated high expression in stems. *IbPLAIIγ*, *IbPLAIIπ*, and *IbPLAIIζ* were highly expressed in petals, whereas *IbPLAIIε* exhibited high expression in anthers. Five genes, namely *IbPLAIIβ*, *IbPLAIIλ*, *IbPLAIIμ*, *IbPLAIIψ*, and *IbPLAIIξ*, were prominently expressed in filaments. *IbPLAIIκ* and *IbPLAIIδ* showed the highest expression in the stigma, *IbPLAIIσ* in the ovary, and *IbPLAIIρ* in sepals.

## 4. Discussion

Conventional breeding methods, including genealogy, backcrossing, compound hybridization, and rotational selection, have yielded numerous high-performing crop hybrids adaptable to diverse ecological environments [44]. However, traditional genetic improvement has consistently faced the challenge of lengthy breeding cycles. Double haploid (DH) breeding, accomplished through in vivo haploid induction, provides a mean to develop pure and uniform plants, significantly reducing the time required for breeding new crop varieties [45].

In this study, we characterized *DMP* and *MTL* genes in sweetpotato using bioinformatics analysis methods. We identified and compared these candidate genes with those reported in other species. Furthermore, we predicted the potential functions of these genes by analyzing their three-dimensional protein structures, investigating protein–protein interaction networks, and assessing tissue-specific differential expression patterns.

### 4.1. Identification and Characterization of the DMP Gene Family and Its Potential HI Genes in Sweetpotato

In maize, the quantitative trait locus (qhir8) is a crucial determinant in facilitating high-frequency haploid induction [46], with the associated pathogenic allele for haplotype induction being *ZmDMP*. *ZmDMP* exhibits high expression in pollen, and its homologous genes demonstrate a high level of conservation across various plant species. The knockout of the *DMP* gene induces haploid production in diverse plants, such as maize and Arabidopsis, and this induction is linked to amino acid mutations in transmembrane structures [38].

In this study, we identified 5 *DMP* genes in sweetpotato. Unlike maize (15), rice (18), and *Arabidopsis* (9), *IbDMPs* do not have a subfamily I. Phylogenetic tree analysis revealed that *IbDMP5* is closely associated with haploid-induced genes in *Arabidopsis* (*AtDMP8*, *AtDMP9*) and maize (*ZmDMP*), placing it within the same branch (Figure 2B). Protein ML phylogenetic tree analysis and examination of gene conserved structures demonstrated that *IbDMP5* exhibits a close evolutionary relationship with *ZmDMP* homologous genes across multiple species; it belongs to subfamily IV and shares a highly similar conserved gene structure (Figure 4A and Figure 5A). The genes within the *IbDMP2–IbDMP5* cluster display a straightforward structure, with only *IbDMP5* containing an intron, implying a propensity for conserved functionality.

Within the promoter region of *IbDMP5*, a *cis*-acting regulatory element associated with seed-specific regulation was identified (Figure 6A), hinting at a potential involvement in plant pollination and fertilization processes. Analyzing the three-dimensional protein structure and amino acid sequence revealed a remarkable similarity between the IbDMP5 protein and *DMP* proteins from other species, suggesting a shared function (Figure 7A and Figure S4A). Moreover, *IbDMP2*, *IbDMP3*, *IbDMP4*, and *IbDMP5* each feature 3–4 transmembrane domains (Figure S7), aligning with protein function predictions indicating their participation in regulating the organization of the endomembrane system. Additionally, *IbDMP5* is predicted to play a role in regulating pollen differentiation and fertilization, thereby inducing haploid production (Figure 8A). Analysis of tissue differential expression patterns revealed significant expression of *DMP* genes in reproductive tissues, with *IbDMP5* exhibiting the highest expression in anthers. In conclusion, *IbDMP5* emerges as a potential HI gene within the sweetpotato *DMP* gene family, demonstrating prospects for future development.

### 4.2. Identification and Characterization of the pPLA Gene Family and MTL Genes in Sweetpotato

Stock6 is a naturally occurring HI line in maize, with an induction rate ranging from 2.3% to 3.2% [47]. The pollen-specific phospholipase gene (*MTL/ZmPLA1/NLD*), derived from qhir1, is a HI gene that contributes to the formation and development of maize pollen as well as the elongation of pollen tubes. *MTL/ZmPLA1/NLD* is a member of the *pPLA* family, a subgroup within the phospholipase A family [48]. The majority of studies on *MTL* genes have focused on monocots, investigating haploid induction in crops through the knockout of the homologous genes of *MTL/ZmPLA1/NLD*.

In sweetpotato, we identified 25 members of the *pPLA* family, classified into three subfamilies (Figure 2B). The *MTL* gene in maize and the *MTL* homologous genes reported in rice, wheat, and millet belong to the *pPLAII* subfamily. The *IbPLAII* subfamily comprises 16 members, and in comparison with other subfamilies, pPLAII exhibits a distinct Motif7 structure, potentially linked to the subfamily’s function (Figure 3B). ML protein developmental tree analysis showed that *IbPLAIIκ*, *IbPLAIIλ*, and *IbPLAIIμ* were in the same clade as other *MTL* homologous genes (Figure 4B). Conserved structure analysis showed that *IbPLAIIκ* only lacked the Motif12 structure (Figure 5B) compared with the *MTL* homologous gene in monad, which may be related to monodicot differences. In addition, it is worth noting that the 2000 bp promoter region upstream of the *IbPLAIIκ* gene does not contain response elements related to reproductive development, but seed-specific response elements (Figure 6B) were found in other members of the *pPLAII* family, suggesting that there may be redundancy in the function of the *pPLAII* subfamily in regulating plant reproductive development. Protein tertiary structure and amino acid sequence analysis showed that *IbPLAIIκ* and *ZmMTL* may have close homology (Figure 7B and Figure S4B).

Protein–protein interaction network analysis showed that the *pPLAII* subfamily regulates the lipid catabolism process (Figure 8B), which may be related to the development of plant anther cuticle and pollen wall formation [49]. Expression pattern analysis indicated high expression of most *pPLAII* subfamily members in reproductive tissues. Specifically, *pPLAIIκ*, *pPLAIIλ*, and *pPLAIIμ* exhibited elevated expression solely in anthers, filaments, and stigma, suggesting their potential role as *MTL* homologous genes in sweetpotato. Nevertheless, considering the lack of conservation of *MTL* in dicots and the potential functional redundancy of the *pPLARII* family, the complete exclusion of the potential haploid induction function of other members is not warranted. For example, in the dicot *Arabidopsis*, *AtpPLAIIλ*—which is closely related to the evolution of *MTL* homologous genes in *ZmMTL* and other species—does not exhibit the haploid induction function [18]. Further gene editing of these potential haploid genes is necessary to validate their haploid-induced function.

## 5. Conclusions

Sweetpotato is a crucial resource for food, feed, and industrial applications [50]. According to the Food and Agriculture Organization of the United Nations (FAO, 2022), China, with a planted area of 2.206 million hectares and a production of 447.8 million tons in 2021, holds the title of the world’s leading sweetpotato producer. The high-yield production of sweetpotato serves as a robust assurance for food security in both China and globally [51]. Nevertheless, threats such as environmental degradation, arable land salinization, and rising instances of pests and diseases pose serious challenges to sweetpotato yields and quality. Concurrently, the world’s growing population is exacerbating the global food crisis. Cultivating new varieties with ideal traits, including high yield, disease resistance, and nutritional value, is essential for the sustainable development of agriculture. Generally, employing modern genomic tools and biotechnology to establish an efficient and feasible breeding system represents the optimal approach for overcoming these obstacles. Double haploid technology, capable of obtaining pure strains with excellent traits within two generations, stands out as a crucial method for enhancing breeding efficiency [52]. Haploid induction serves as the pivotal process for actualizing haploid breeding. The development of key HI genes, such as *ZmPLA1/MATL/NLD* and *ZmDMP* in maize, has significantly contributed to the advancement of haploid technology systems [53]. The induction of haploids spans monocotyledons like rice, wheat, and barley, and dicotyledons—including *Arabidopsis*, tomato, potato, and tobacco [54]. Additionally, manipulation of *CENH3* and its cognate genes has demonstrated the induction of haploids. With further research, several potential HI genes have been identified, including *ZmPLD3*, *ECS1/2* [55], *OsBBM1* [56], and *KNL2* [57]. Knockdown of multiple HI genes in the same plant can act synergistically to increase the HIR. In maize, the haploid induction rate had a 5- to 6-fold increase for the zmpla1zmdmp double mutation compared with the zmpla1 single mutation [5]. Similarly, knockdown of *ZmPLD3* in the zmpla1 background increased haploid induction in the mutant. However, this synergy may be related to species specificity. Manipulating single or multiple HI genes in sweetpotato may be the most suitable approach for creating DH lines.

Gene editing in hexaploid plants often faces inefficiency. We developed an efficient gene-editing system by constructing an expression vector based on the replicon of the sweetpotato leaf curl virus (SPLCV) [58]. Additionally, we bolstered resistance in sweetpotato using the CRISPR-Cas13 system, thereby enhancing the efficiency of breeding for resistance against sweetpotato virus diseases (SPVDs) [59]. The establishment of an efficient root transgenic system further simplified the creation of transgenic sweetpotato strains [32], which has provided a technical foundation for generating double haploid strains.

In conclusion, this study was the first genome-wide identification and characterization of HI genes in the sweetpotato *DMP* gene family and *pPLA* gene family. A total of 5 *DMP* genes and 25 *PLA* genes were identified in sweetpotato. *IbDMP5*, *IbPLAIIκ*, *IbPLAIIλ*, and *IbPLAIIμ* were identified as potential HI genes in sweetpotato through phylogenetic tree analysis, gene structure analysis, conserved Motif and domain analysis, promoter element analysis, protein three-dimensional structure modeling and amino acid sequence alignment analysis, protein–protein interaction regulatory network analysis, and tissue differential expression pattern analysis (Table 1). These findings offer crucial genetic insights into haploid induction in sweetpotato and aid in the development of double haploid (DH) lines in this crop.

## Figures and Tables

**Table 1:** Characterization of haploid inducer genes in sweetpotato.

Name	Gene Locus ID	AA	MW (Da)	pI	Instability Index	Aliphatic Index	Gravy	Subcellular Localization
*IbDMP5*	g47273.t1	225	24,123	8.19	30.38	87.87	0.341	plas: 4, E.R.: 4, cyto: 3, nucl: 2, vacu: 1
*IbPLAⅡ* *κ*	g29928.t1	412	45,105	9.08	29.87	87.48	−0.177	chlo: 11, nucl: 2, vacu: 1
*IbPLAⅡ* *λ*	g38666.t1	1226	136,725	6.58	40.92	91.14	−0.282	nucl: 3.5, chlo: 3, plas: 3, cyto_nucl: 3, cyto: 1.5, mito: 1, E.R.: 1, golg: 1
*IbPLAⅡ* *μ*	g38668.t1	292	32,779	8.25	46.3	87.47	−0.305	chlo: 4, nucl: 3, cyto: 3, cysk: 2, plas: 1, E.R. vacu: 1

MW, molecular weight; pI, isoelectric point; GRAVY, grand average of hydropathicity.

## Data Availability

The data presented in this study are available on request from the corresponding author.

## Supplementary Materials

The following supporting information can be downloaded at: https://www.mdpi.com/article/10.3390/genes15030354/s1, Figure S1: Size of sweetpotato bud; Figure S2: Microscopic images of pollen microstructure [60]; Figure S3: Genetic identification of the Venn diagram, (A): IbPLAs; (B): IbDMPs; Figure S4: Comparison of amino acid sequences of haploid-inducible genes, (A): IbCENH3; (B): IbDMPs; (C): IbMTLs; (D): IbPLDS; Figure S5: Syntenic analysis of pPLAs and DMPs; Figure S6: Prediction of transmembrane structure of DMPs; Figure S7: Tissue expression patterns analysis of IbPLA genes in sweetpotato; Table S1: The amino acid sequences of DMPs and pPLAs; Table S2: Characterization of DMP and pPLA genes in sweetpotato; Table S3: *Cis*-acting regulatory elements present in the promoter regions of DMP and pPLA genes of sweetpotato; Table S4: The primers list used for qRT–PCR.
