# Supplementary material for: Genome-Wide Identification and Expression Analysis of the DMP and MTL Genes in Sweetpotato (Ipomoea batatas L.)
Source: Genes (Basel). 2024 Mar 12;15(3):354. doi: 10.3390/genes15030354 (PMC10970459; doi:10.3390/genes15030354)
Supplement: Supplementary file 1 [file genes-15-00354-s001.zip › FigS1-S7.pdf]

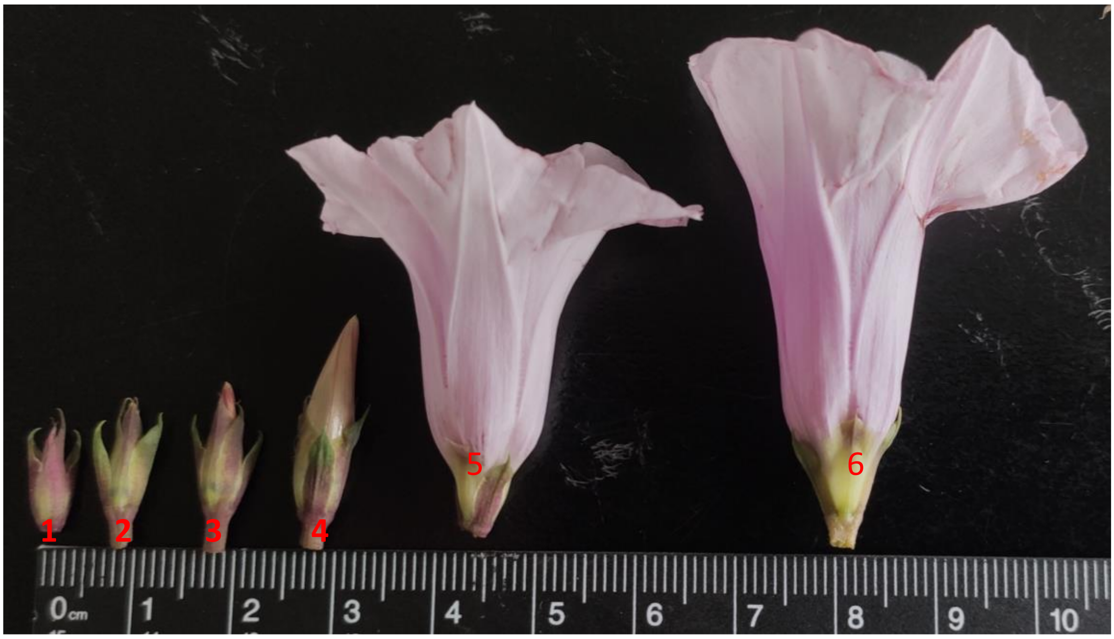

Figure S1. Size of sweetpotato bud.

The pollens of No.1 and No.2 were selected as immature anthers, and No.5 and No.6 were mature anthers.

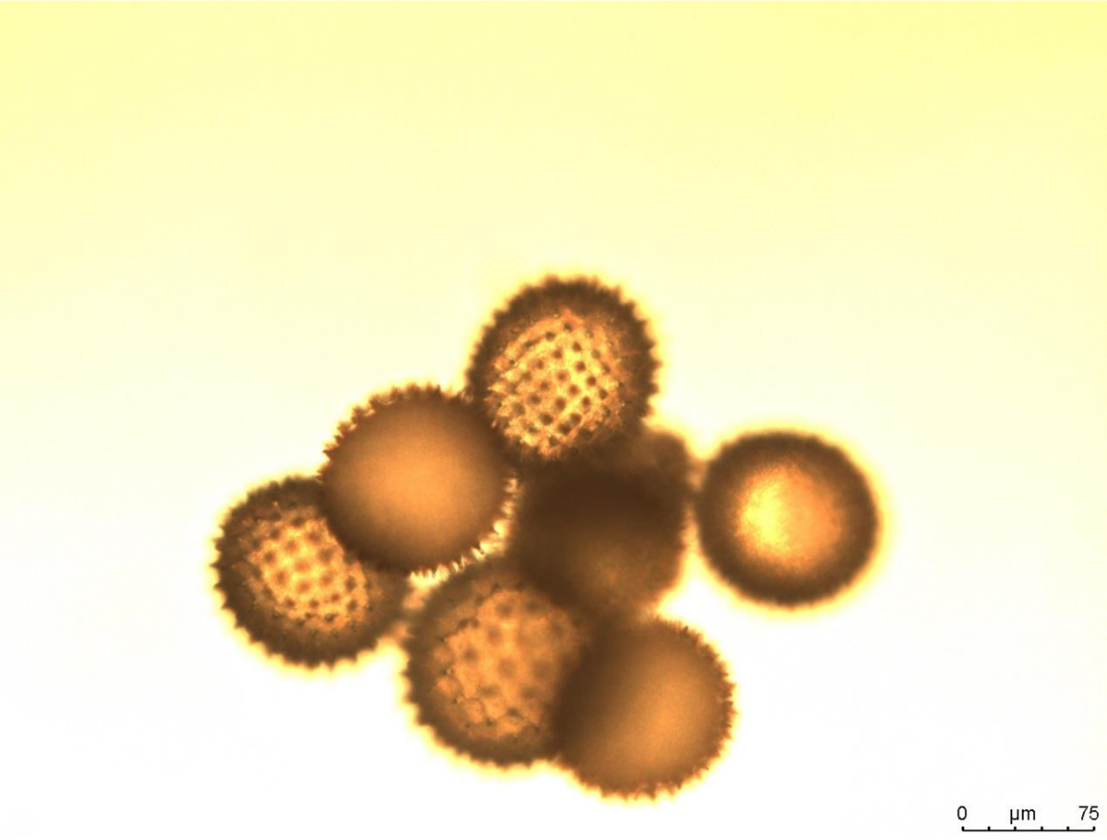

Figure S2. Microscopic images of pollen microstructure.

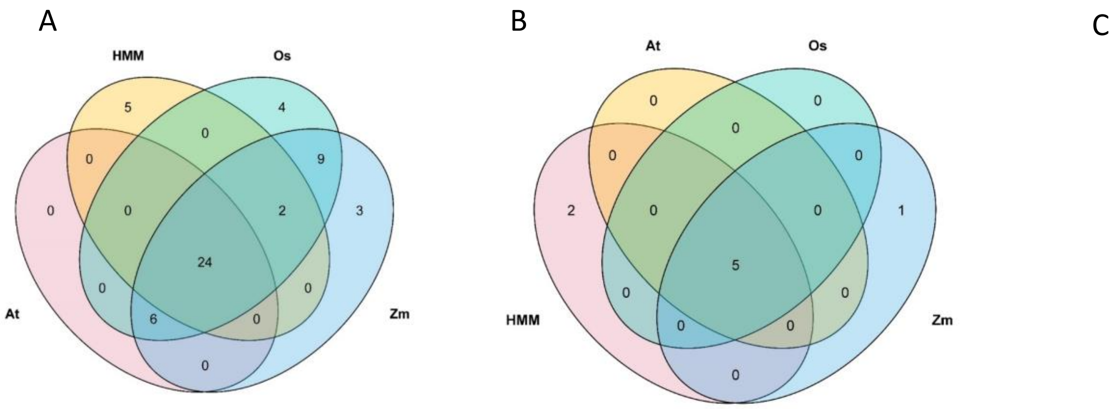

Figure S3. Genetic identification of the Venn diagram.

A： IbPLAs B: IbDMPs

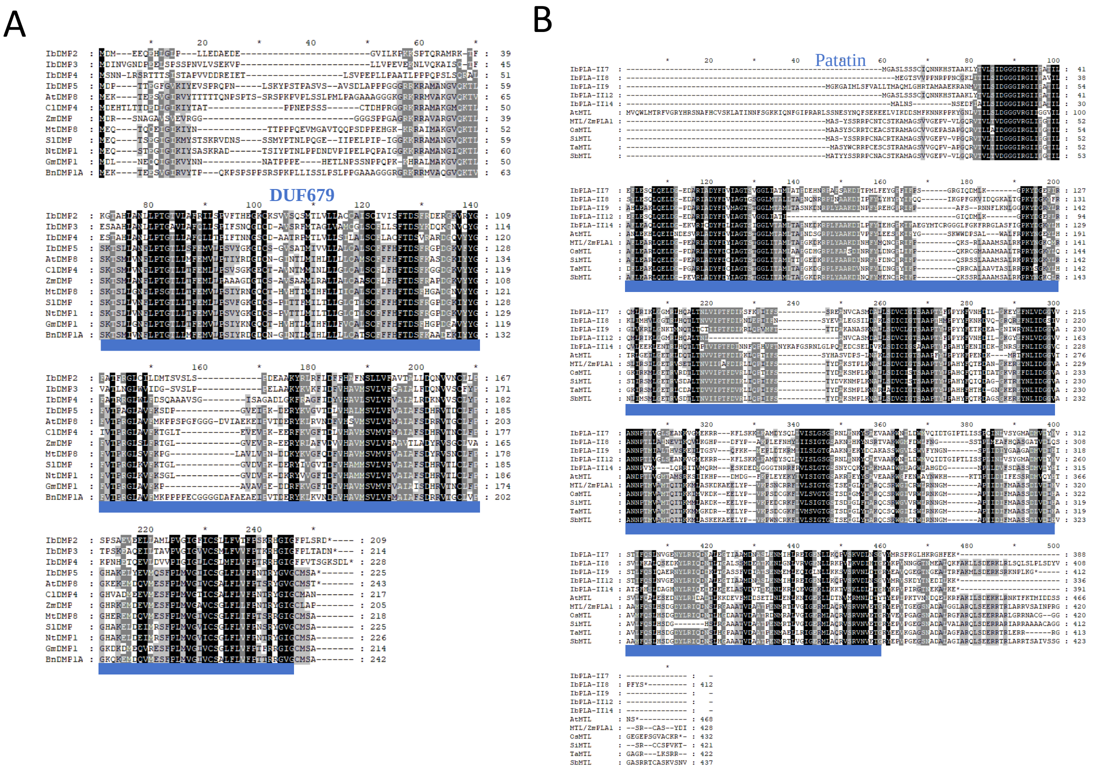

Figure S4. Comparison of amino acid sequences of haploid-inducible genes.

A： IbCENH3 B: IbDMPs C: IbMTLs D: IbPLDS

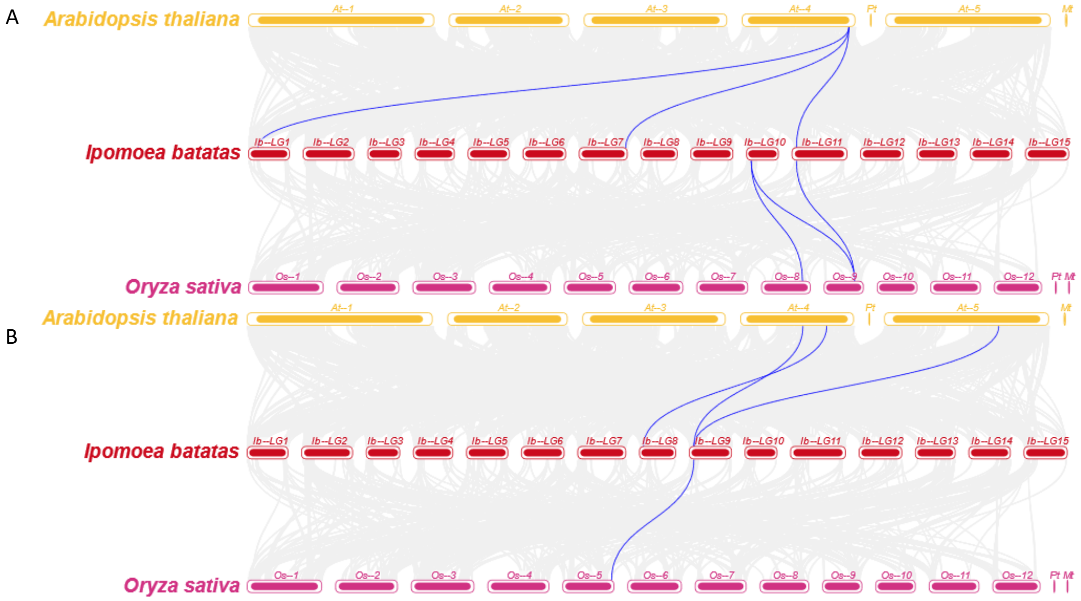

Figure S5. Syntenic analysis of pPLAs and DMPs.

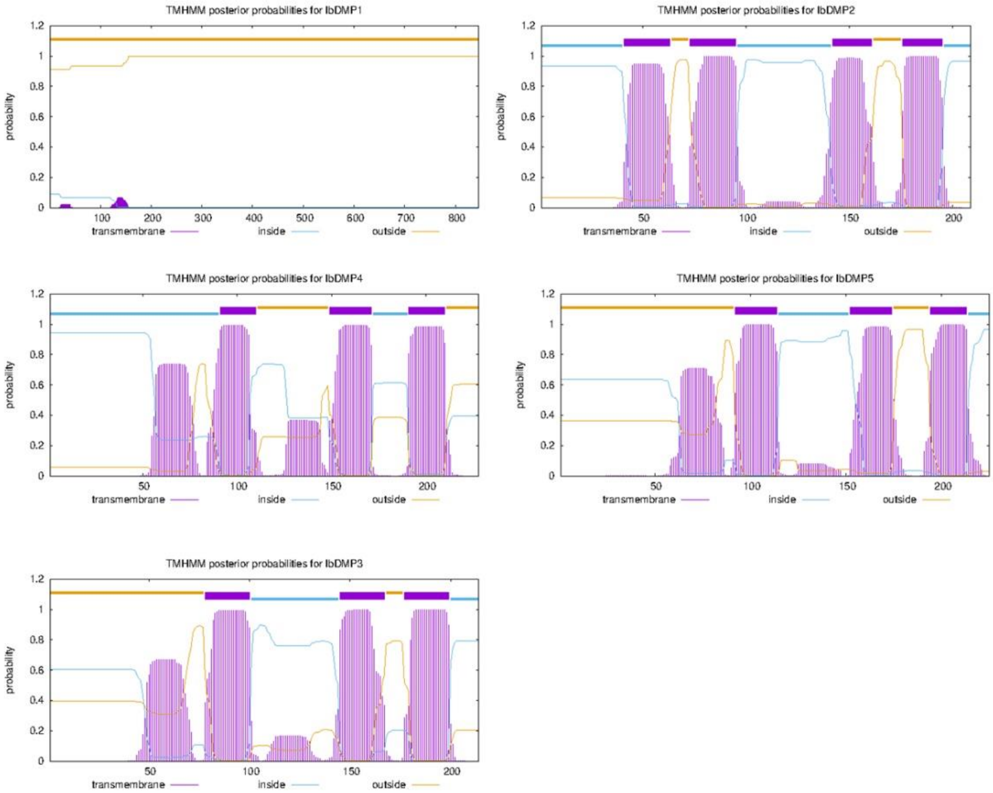

Figure S6. Prediction of transmembrane structure of DMPs.

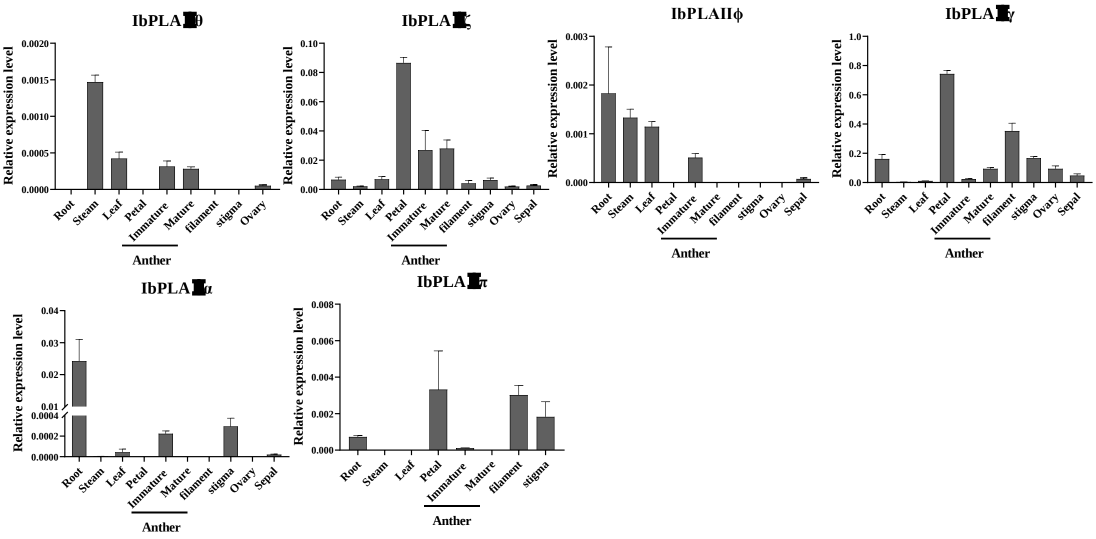

Figure S 7 Tissue expression patterns analysis of IbPLAs genes in sweet potato**.**
